# Menopause is associated with postprandial metabolism, metabolic health and lifestyle: The ZOE PREDICT study

**DOI:** 10.1016/j.ebiom.2022.104303

**Published:** 2022-10-18

**Authors:** Kate M. Bermingham, Inbar Linenberg, Wendy L. Hall, Kirstin Kadé, Paul W. Franks, Richard Davies, Jonathan Wolf, George Hadjigeorgiou, Francesco Asnicar, Nicola Segata, JoAnn E. Manson, Louise R. Newson, Linda M. Delahanty, Jose M. Ordovas, Andrew T. Chan, Tim D. Spector, Ana M. Valdes, Sarah E. Berry

**Affiliations:** a Department of Twins Research and Genetic Epidemiology, King's College London, London, UK; b Zoe Ltd, London, UK; c Department of Nutritional Sciences, King's College London, London, UK; d Department of Clinical Sciences, Lund University, Malmö, Sweden; e Department of Nutrition, Harvard Chan School of Public Health, Boston, MA, USA; f Department CIBIO, University of Trento, Trento, Italy; g Department of Medicine, Brigham and Women's Hospital, Harvard Medical School, Boston, MA, USA; h Newson Health Research and Education, Stratford-upon-Avon, UK; i Diabetes Center, Department of Medicine, Massachusetts General Hospital, Boston, MA, USA; j Harvard Medical School, Boston, MA, USA; k JM-USDA-HNRCA at Tufts University, Boston, MA, USA; l IMDEA Food Institute, CEI UAM + CSIC, Madrid, Spain; m UCJC, Madrid, Spain; n Clinical and Translational Epidemiology Unit, Massachusetts General Hospital, Boston, MA, USA; o School of Medicine, University of Nottingham, Nottingham, UK; p Nottingham NIHR Biomedical Research Centre, Nottingham, UK

**Keywords:** Menopause, Postprandial metabolic responses, Age-matched subgroups

## Abstract

**Background:**

The menopause transition is associated with unfavourable alterations in health. However, postprandial metabolic changes and their mediating factors are poorly understood.

**Methods:**

The PREDICT 1 UK cohort (*n*=1002; pre- *n*=366, peri- *n*=55, and post-menopausal females *n*=206) assessed phenotypic characteristics, anthropometric, diet and gut microbiome data, and fasting and postprandial (0–6 h) cardiometabolic blood measurements, including continuous glucose monitoring (CGM) data. Differences between menopausal groups were assessed in the cohort and in an age-matched subgroup, adjusting for age, BMI, menopausal hormone therapy (MHT) use, and smoking status.

**Findings:**

Post-menopausal females had higher fasting blood measures (glucose, HbA1c and inflammation (GlycA), 6%, 5% and 4% respectively), sugar intakes (12%) and poorer sleep (12%) compared with pre-menopausal females (*p*<0.05 for all). Postprandial metabolic responses for glucose_2hiauc_ and insulin_2hiauc_ were higher (42% and 4% respectively) and CGM measures (glycaemic variability and time in range) were unfavourable post- *versus* pre-menopause (*p*<0.05 for all). In age-matched subgroups (*n*=150), postprandial glucose responses remained higher post-menopause (peak_0-2h_ 4%). MHT was associated with favourable visceral fat, fasting (glucose and insulin) and postprandial (triglyceride_6hiauc_) measures. Mediation analysis showed that associations between menopause and metabolic health indicators (visceral fat, GlycA_360mins_ and glycaemia (peak_0-2h_)) were in part mediated by diet and gut bacterial species.

**Interpretation:**

Findings from this large scale, in-depth nutrition metabolic study of menopause, support the importance of monitoring risk factors for type-2 diabetes and cardiovascular disease in mid-life to older women to reduce morbidity and mortality associated with oestrogen decline.

**Funding:**

Zoe Ltd.

**Research in context**

**Evidence before this study**

We searched PubMed for articles published up to Feburary 4th, 2022 using the terms “menopause metabolic syndrome”, “menopause postprandial responses”, “menopause gut microbiome”, “menopause diet quality” and “menopause inflammation”. We found several published observational studies showing increases in body mass index, visceral fat, cardiometabolic risk and inflammatory markers following menopause. Changes in gut microbiome relating to oestrogen levels and menopausal status have also been reported, and a weak or non-existent link between menopausal status and insulin resistance has been published before. The role of dietary intake and changes in habitual diet in all of the above have not been explored to date nor has the proportion of time that pre-, peri- and post-menopausal women are within healthy glycaemic ranges (Time spent in range (TIR)). There are several studies that have investigated post-menopausal changes on postprandial metabolic responses but only small studies (*n*<100) have sought to separate the effect of age from the effect of menopause from postprandial responses, typically focusing on only one component of the postprandial response. Assessments of which factors are mediating the metabolic changes observed during menopause have not yet been performed in deeply phenotyped large cohorts.

**Added value of this study**

In this deeply phenotyped cohort, we show that continuous measures of glycaemic control (TIR and glycaemic variability) are unfavourable post- *versus* pre-menopause. Using an age-matched design we report that higher glycaemic postprandial responses are not due to age. We also report a protective association between menopausal hormone therapy and visceral fat and fasting and postprandial measures. Using formal mediation analysis we find that associations between menopausal status and key metabolic health indicators (visceral fat, inflammatory and glycaemic postprandial responses) are in part mediated by diet quality and gut bacterial species abundances. We also confirm differences in body composition, fasting blood measures, postprandial metabolites, lifestyle, diet, microbiome, sleep and mood across sex, age and menopausal status.

**Implications of all the available evidence**

Postprandial glycaemic responses worsen in the post-menopausal state, not because of increasing age but because of altered hormonal status. These effects are in part mediated by changes in dietary habits and gut microbiome composition indicating these two modifiable factors are potential targets to reduce the morbidity and mortality associated with loss of oestrogen. These findings will be of high importance to clinicians, researchers, the general public and policy makers, enabling stratified dietary and lifestyle advice to attenuate the unfavourable effects of menopause on cardio-metabolic risk.

Alt-text: Unlabelled box

## Introduction

Women spend more than one-third of their lives in a post-menopausal state. Menopause is the definitive disappearance of menstruation due to the depletion of ovarian activity, occurring after 12 months of amenorrhoea.^1^ The menopause transition, also known as perimenopause, is the beginning of menstrual irregularities when symptoms of female sex hormone deficiency begin. Menopausal change is associated with higher prevalence of metabolic syndrome and cardiovascular risk factors, as well as alterations in mood, sleep, diet and other lifestyle factors.^2^, ^3^, ^4^ However, whether the reported changes associated with menopause are due to hormonal alterations, psychological changes associated with the transition, natural ageing, social and behavioural factors of midlife or genetic vulnerability is less clear and warrants further exploration.

Extensive evidence shows that changes in body composition including loss of lean body mass, accumulation of fat mass and redistribution of the adipose tissue in the abdominal area occur with menopause.^5^ Subsequently, unfavourable fasting blood measures^3^ and a shift to an atherogenic lipid profile (increases in total cholesterol, low-density lipoprotein cholesterol (LDL-C), and apolipoprotein B) occur independently of age, due to menopause.^2^ However, less is known regarding the impact of menopause on the integrated postprandial metabolic response.^6^ Given that humans spend the majority of their day in the postprandial (1–8 h post eating) phase (∼18 h/d) and postprandial lipaemia and glycaemia are independent risk factors for cardiovascular diseases (due to their downstream effects on inflammation, oxidative stress, haemostatic function and lipoprotein remodelling), studies exploring multi-factorial postprandial responses with respect to menopausal status are needed. Furthermore, the gut microbiome is increasingly recognised as an important regulator of metabolism and is associated with multiple cardiometabolic risk factors.^7^^,^^8^ Whilst alterations in gut microbiome composition have been shown during the menopausal transition, its role in increased metabolic risk faced by menopausal women remains unclear.^8^

In light of the well-recognised changes that occur in lifestyle and body composition upon menopause, research furthering our understanding of the key metabolic and microbial changes occurring in concert may help provide tailored lifestyle and dietary advice for women during their menopausal transition and post-menopause. This study leveraged the densely phenotyped ZOE PREDICT cohort to, firstly, characterise lifestyle, diet and health measures in pre-, peri- and post-menopausal women and, secondly, explore the physiological changes of menopause with a focus on postprandial metabolism and the gut microbiome. We report; 1) differences in body composition, fasting blood measures, postprandial metabolites, lifestyle, diet, microbiome and mood across sex, age and menopausal status, 2) an independent association of menopause with postprandial glucose responses in an age-matched subgroup, 3) a protective association between menopausal hormone therapy (MHT) use and visceral fat and fasting and postprandial measures, and 4) a mediation effect of diet and bacterial species on visceral fat and inflammation, by menopause status.

## Methods

### Study design and population

The ZOE PREDICT 1 study investigated the effect of foods and individual characteristics on postprandial response variability in a randomized single-arm, single-blinded and multicentre intervention design, as described previously.^9^^,^^10^ Briefly, PREDICT 1 enrolled 1102 healthy adults aged 18–65 years between June 2018 and May 2019, of which 1002 participated in the UK and 100 in the US. Participants took part in a 14-day intervention, on the first day of which they completed a clinical visit at St. Thomas’ Hospital with baseline measures and a controlled test meal challenge. This was followed by 13 days of an at-home intervention with standardized test meal challenges of various nutritional content (**Supplementary Table 1**), as well as the recording of all ad libitum free-living intake of foods and drinks through a specially designed study mobile phone application. Participants wore digital devices to measure their physical activity and sleep duration and quality (wrist-based accelerometer AX3, Axivity, Newcastle-Upon-Tyne, UK) and their interstitial blood glucose via continuous glucose monitor placed on the upper arm (CGM; Abbott Freestyle Libre, Germany). Primary outcomes are reported elsewhere^9^ and include gut microbiome profile, blood lipids and glucose, sleep, physical activity and hunger and appetite assessment. This is a secondary analysis to the previously published primary analysis, further details including sample size calculations can be found elsewhere.^9^^,^^10^ Data for this secondary analysis in pre-, peri- and post-menopausal females from PREDICT UK only is reported in this paper. Pre-established exclusion criteria for PREDICT 1 were Type-2 diabetes and at the analysis level for this secondary analysis, participants were excluded if they experienced early menopause defined as the onset of menopause at the age of 40 years or below.

### Ethical approval

The PREDICT 1 study was ethically approved by the UK Research Ethics Committee and Integrated Research Application System (IRAS 236407) and a US institutional review board (Partners Healthcare IRB 2018P002078). The trial was run according to the Declaration of Helsinki and Good Clinical Practice and registered on ClinicalTrials.gov (NCT03479866). Participants provided informed written consent before taking part in the study.

### Ascertaining menopausal status

Participants completed a health and lifestyle questionnaire prior to their baseline visit (amended Twins Research health and lifestyle questionnaire). Minor modifications were made to this questionnaire to conform to the US study population. Alongside medical history and the use of menopausal hormone therapy, the questionnaire ascertained menopausal status with the question “what is your menopausal status?” and the answer options pre-menopause, perimenopause, post-menopause (defined as amenorrhea for at least 12 months) and unsure. Participants were also asked “How old were you when you became post-menopausal (when you stopped having periods for one year or more)?”. A total of 627 women specified their menopausal status by questionnaire and completed their baseline clinical measures, PREDICT 1 (*n*=366, *n*=55, *n*=206 respectively).

Information on outcome measurements, standardised test meal challenges, food and mood data, physical activity, microbiome samples, and CGM devices are in the Supplementary Materials. For sleep, participants completed the Pittsburgh Sleep Quality Index (PSQI) questionnaire, a validated tool measuring self-reported sleep quality and sleep disturbance on a scale from 0 to 21, where higher scores indicate worse sleep quality.

### Statistics

#### Basic analysis

All statistical analyses were performed using R statistical suite version 1.3.1093. The descriptive characteristics, including baseline characteristics, fasting blood biomarkers, lifestyle, diet, and mood data for participants (*n*=1002) are summarised in Table 1. Data were tested for normality and transformed where applicable using log or sqrt transformation. Differences in participant characteristics were assessed between males and females and between menopausal groups (pre- *v*s. post-menopause and pre- *v*s. perimenopause) using analysis of covariance (ANCOVA) and logistic regression for continuous and categorical data, respectively. Differences between sexes were adjusted for age and BMI (when testing anthropometric traits only age was included), differences between menopausal groups were adjusted for age, BMI, MHT use and smoking status. Mood data was additionally adjusted for any history of mental health. Partial spearman's correlations were performed to investigate the relationship between age and outcome measures in males and females separately while adjusting for BMI using the “*ppcor”* package. Significant correlations with age were retested in a subcohort of females of equal sample size to the male cohort, 20 random sample subgroups were tested for each variable and the average correlation coefficient and p-value was selected. Inter-individual variation was measured using the coefficient of variation (CV%; s.d./mean). The Benjamini-Hochberg correction for multiple comparisons was applied.^11^ Statistically significant thresholds were based on false discovery rate (FDR) cut-offs (*q*< 0.05).

**Table 1:** Characteristics of the PREDICT 1 cohort across sex.

	Total PREDICT 1 cohort		Males			Females			Males vs Females
	Mean (SD)	n	Mean (SD)	n	Corr age (r)	Mean (SD)	n	Corr age (r)	*p*-value
Age (years)	45.6 (12.0)	(1000)	43.4 (12.3)	(274)	-	46.5 (11.8)	(726)	-	0.00***
Systolic BP (mm/Hg)	124 (14.6)	(987)	130 (12.5)	(270)	0.09	122 (14.9)	(717)	0.35***	0.00***
Diastolic BP (mm/Hg)	75.8 (10.2)	(987)	77.3 (10.1)	(270)	0.28***	75.3 (10.2)	(717)	0.16***	.0.00**
**Body composition**									
Height (cm)	169 (9.02)	(1000)	178 (7.56)	(274)	−0.12	165 (6.62)	(726)	−0.11**	0.00***
Weight (kg)	72.8 (15.4)	(1000)	82.6 (14.1)	(274)	0.16*	69.2 (14.3)	(726)	0.19***	0.00***
BMI (kg/m^2^)	25.6 (5.06)	(1000)	26.02 (4.18)	(274)	0.30***	25.4 (5.34)	(726)	0.25***	0.00**
Visceral fat mass (g)	527 (311)	(917)	595 (286)	(256)	0.58***	501 (316)	(661)	0.45***	0.00***
Waist: Hip ratio	0.84 (0.08)	(1000)	0.91 (0.08)	(274)	0.41***	0.82 (0.07)	(726)	0.29***	0.00***
**Fasting blood markers**									
Glucose (mmol/L)	4.97 (0.52)	(1000)	5.07 (0.50)	(274)	0.28***	4.93 (0.52)	(726)	0.27***	0.00***
Triglycerides (mmol/L)	1.06 (0.54)	(1000)	1.19 (0.62)	(274)	−0.01	1.01 (0.49)	(726)	0.11**	0.00***
Insulin (mIU/L)	6.17 (4.28)	(1000)	6.46 (4.74)	(274)	−0.06	6.06 (4.09)	(726)	−0.09*	0.82
GlycA (mmol/L)	1.33 (0.18)	(999)	1.37 (0.18)	(274)	−0.05	1.32 (0.18)	(725)	0.09*	0.00**
Cholesterol (mmol/L)	4.95 (0.99)	(1000)	4.94 (1.02)	(274)	0.27***	4.95 (0.97)	(726)	0.40***	0.59
LDL (mmol/L)	2.93 (0.79)	(984)	3.06 (0.77)	(272)	0.20	2.88 (0.79)	(712)	0.42***	0.00***
Non-HDL (mmol/L)	3.29 (1.01)	(1000)	3.48 (1.04)	(274)	0.17*	3.22 (0.99)	(726)	0.32***	0.00***
HbA1c (%)	5.47 (0.32)	(998)	5.48 (0.28)	(274)	0.16*	5.47 (0.34)	(724)	0.35***	0.57
Quicki score	0.38 (0.06)	(1000)	0.38 (0.06)	(274)	0.00	0.38 (0.06)	(726)	0.04	0.64
HOMA-IR	1.40 (1.12)	(1000)	1.49 (1.29)	(274)	0.00	1.36 (1.05)	(726)	−0.04	0.71
ASCVD 10y risk	0.02 (0.03)	(435)	0.04 (0.05)	(128)	-	0.02 (0.02)	(307)	-	0.71
Liver Fat Probability score	0.17 (0.14)	(927)	0.21 (0.14)	(259)	−0.16*	0.15 (0.13)	(668)	0.08	0.00***
**Postprandial markers**									
Glucose_2hiauc_ (mmol/L.s)	7409 (5033)	(904)	6925 (4533)	(255)	0.10	7599 (5207)	(649)	0.21***	0.34
Insulin_2hiauc_ (mIU/L.s)	268,884 (170,331)	(904)	263,820 (165,109)	(254)	−0.05	270,863 (172,414)	(650)	−0.06	0.12
Triglyceride_6hiauc_ (mmol/L.s)	10,372 (7580)	(810)	12,409 (8537)	(232)	−0.15	95,545 (7004)	(578)	0.22***	0.00***
GlycA_6rise_	−0.02 (0.03)	(957)	−0.03 (0.03)	(267)	0.08	−0.02 (0.03)	(690)	0.03	0.00***
**Lifestyle & diet**									
Sleep (PSQI, 0-21)	6.88 (2.25)	(911)	6.74 (2.13)	(257)	−0.05	6.94 (2.3)	(654)	0.07	0.28
Activity level (0-5)	3.83 (1.6)	(969)	4.03 (1.53)	(268)	-	3.75 (1.62)	(701)	-	0.64
Diet (HEI, 0-100)	58.2 (10.6)	(928)	56.6 (11.5)	(262)	0.10	58.8 (10.2)	(666)	−0.03	0.03*
Energy (kcal)	1685 (497)	(928)	1823 (549)	(262)	−0.02	1632 (466)	(666)	0.08	0.00***
Carbohydrate (% energy)	44.0 (8.21)	(928)	43.6 (8.74)	(262)	−0.02	44.1 (7.99)	(666)	-0.13**	0.20
Protein (% energy)	18.0 (3.36)	(928)	17.9 (3.41)	(262)	−0.01	18.1 (3.34)	(666)	0.07	0.63
Fat (% energy)	37.1 (6.49)	(928)	37.3 (6.95)	(262)	−0.09	37.1 (6.31)	(666)	0.01	0.74
Sugars (g)	85.8 (34.5)	(928)	89.1 (37.0)	(262)	0.07	84.6 (33.4)	(666)	0.08	0.10
**Mood**									
Less accomplished (Yes%)	19.1	(964)	19.0	(268)	-	19.1	(696)	-	0.08
Less concentration (Yes%)	11.4	(958)	13.9	(266)	-	10.4	(692)	-	0.22
Contentedness*	2.96 (1.11)	(965)	2.86 (1.1)	(268)	-	3.00 (1.10)	(697)	-	0.34
Energetic*	3.05 (1.16)	(964)	2.90 (1.10)	(268)	-	3.11 (1.18)	(696)	-	0.20
Sadness*	4.91 (0.98)	(928)	4.94 (0.99)	(268)	-	4.90 (0.98)	(697)	-	0.94
Lower social activity*	4.6 (0.73)	(928)	4.66 (0.73)	(267)	-	4.58 (0.72)	(699)	-	0.03*

*Scoring ranges from lowest “all of the time” to highest “none of the time”. ANCOVA (continuous) and logistic regression (categorical) adjusted for age and BMI. Anthropometric traits were adjusted for age only. All p-values adjusted for multiple testing (FDR<0.05); * *p*<0.05, ** *p*<0.01, ***p<0.001.

#### Independent effect of menopause

Age-matched subgroups of pre- and post-menopausal females (*n*=150) and males (*n*=76) were created from the PREDICT 1 cohort using the “*MatchIt”* package. Participants from all three groups with overlapping ages (range 47–56y) were selected for this sub-analysis. Measures from the baseline characteristics, fasting blood biomarkers, lifestyle, diet, and mood data that were significantly different between pre- and post-menopausal females in the total cohort were carried forward for analysis in the age-matched subgroup. Differences in means between pre- and post-menopausal females were examined with adjustment for age, BMI, MHT use and smoking status. Difference between males versus pre-menopausal females and males versus post-menopausal females were also tested. We also examined the same measures in post-menopausal females using MHT (*n*=35) and post-menopausal females not using MHT (*n*=173) along with a selection of measures with previously reported known associations with MHT (visceral fat, lipids; LDL-cholesterol and postprandial TG).

#### Meal analysis

A selection of set meals was consumed in duplicate (Supplementary Table 1) and the average glycaemic responses (glucose_2hiauc_ and glucose peak_0-2h_) of the two meals were used in analyses where applicable.

#### Mixed effects regression analysis

To determine the independent effect of menopausal status on postprandial metabolic responses mixed effect regression models were performed in the PREDICT 1 cohort. Participants currently using MHT were excluded from this analysis. Data were tested for normality using Shapiro-Wilk and transformed to normality where applicable using log or sqrt transformation. All traits were standardised to have mean 0 and 1 s.d. to allow effect comparison across traits. The model included a postprandial metabolic response as the endpoint variable (Glucose; 30 min concentration, rise (30 min), peak_0-2h_, glucose_2hiauc_, Insulin; 30 min and 360 min concentration, insulin_2hiauc_, TG; 360 min concentration TG_6hrise_, TG_2hiauc_, and GlycA; 360 min concentration, GlycA_4hrise,_ GlycA_6hrise_). Menopausal status, age and BMI were included as fixed effects and family relatedness was also adjusted for as random effect. Two separate models with different covariates were performed to examine the effect of 1) age and 2) age and BMI (FDR *p*<0.05). The same mixed regression models were performed in an age-matched subgroup of pre- and post-menopausal females (*n*=150).

#### Gut microbiome species

Relative abundances of species were examined in pre and post-menopausal females with microbiome data available (*n*=564). The Mann-Whitney U test compared differences in species prevalent at >20% between groups (pre- *v*s. post-menopausal). Statistical significance was considered in the total cohort as FDR *p*<0.20. In the age-matched subgroup (*n*=150) species that were differentially abundant were also examined, and statistical significance was considered as *p*< 0.05.

*Mediation analysis.* The “*Mediation”* package was implemented to test the mediation effects of physical activity, sleep, diet quality and microbiome species (indirect effects) on the total effect of menopausal status on 1) GlycA, 2) glucose peak_0-2h_, and 3) visceral fat, adjusting for age, BMI, MHT use and family-relatedness. Relative abundance of gut microbiome species was used in the mediation analysis and normalised using the arcsine square root transformation as previously described.^9^ We used linear mixed-effects models (“*lme4*” package in R) for both the mediator and outcome models. For all models, we report the percentage causal mediation effect (ACME) and the percentage direct effect (ADE). ACME represents the average size of the effect of menopausal status on 1) GlycA, 2) glucose peak_0-2h_, and 3) visceral fat that is mediated by physical activity, sleep, diet quality or microbiome species, while ADE represents the direct effect of menopausal status on 1) GlycA, 2) glucose peak_0-2h_, and 3) visceral fat.

#### Role of funders

The study sponsors (ZOE Ltd) contributed as part of the Scientific Advisory Board in the study design and collection.

## Results

### The ZOE PREDICT 1 cohort

**Figure 1:**
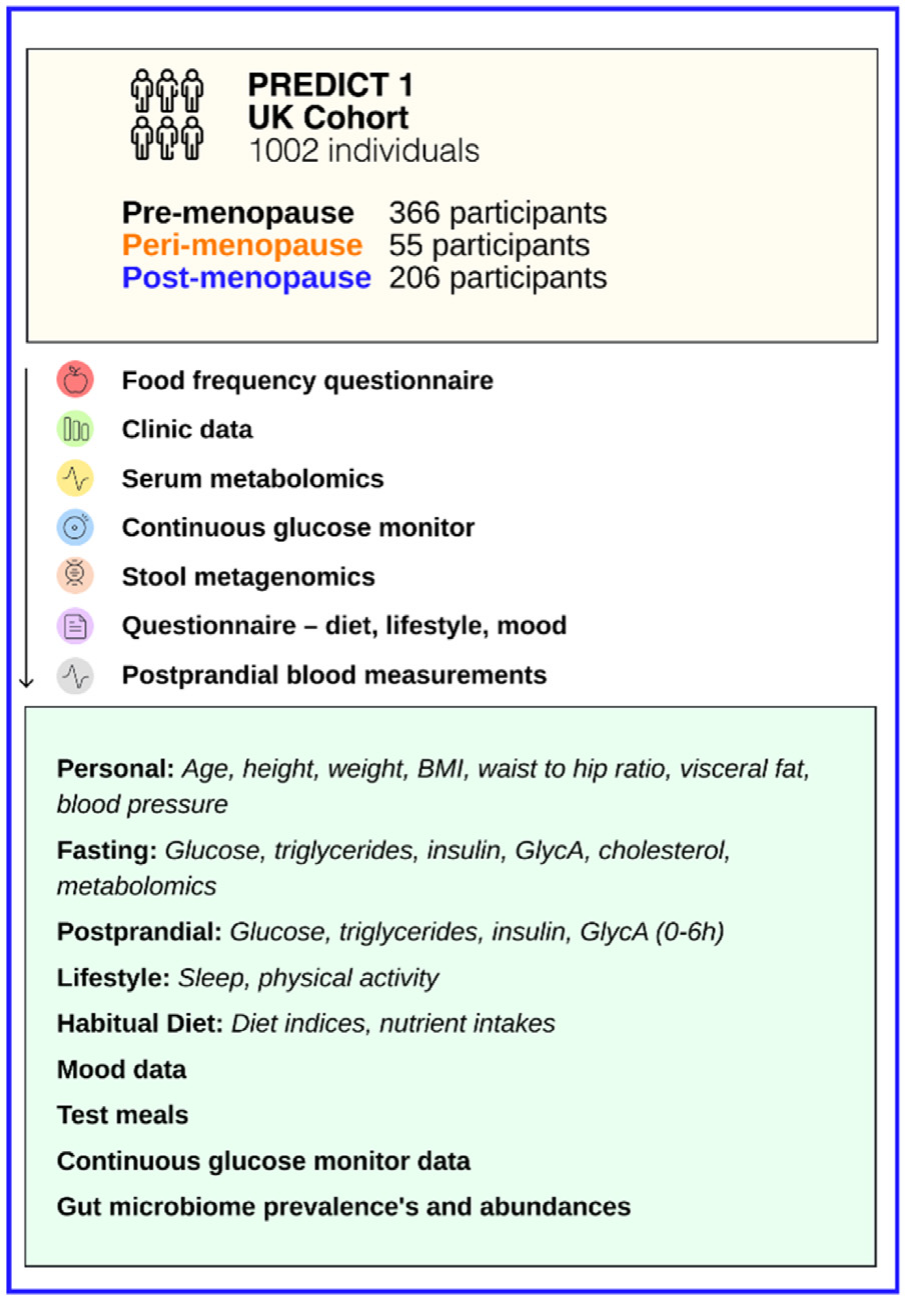
Experimental design. Self-reported pre-, peri- and post-menopausal females were selected from the ZOE PREDICT 1 (UK; *n*=366, *n*=55, *n*=206 respectively) cohort. Phenotypic data were obtained following in-person assessments and during a 13-d at home phase. Personal characteristics, lifestyle factors, diet, fasting and postprandial metabolic response, test meals, continuous glucose levels, gut microbiome composition and mood, were examined across menopausal groups in the PREDICT 1 cohort.

The ZOE PREDICT 1 study collected detailed phenotypic data, habitual diet information, gut microbiome data, and multiple fasting and postprandial cardiometabolic blood marker measurements from 1002 healthy adults from the United Kingdom (NCT03479866), Figure 1. Postprandial measures were collected in the clinic (0–6  h; serum triglyceride (TG), glucose, insulin, glycoprotein acetylation (GlycA) and circulating metabolite (NMR metabolomics panel) concentrations) following sequential mixed-nutrient dietary challenges and remotely (13d) for glucose, using continuous glucose monitors (CGMs) following 8 standardised meals of differing macronutrient content. Self-reported pre-, peri- and post-menopausal females were selected from PREDICT 1 (*n*=366, *n*=55, *n*=206 respectively). Characteristics related to menopausal status including age at menopause, contraception use, MHT use and smoking status are reported in Supplementary Table 2. The study design and the inclusion criteria of study participants are described in detail in the Methods. Further information on research design has been previously published.^9^ Compared to the average UK population, PREDICT 1 participants were older (mean age 46 vs 41 years respectively), had a lower BMI (26 vs 28 kg/m^2^ respectively), were less likely to smoke and the proportion of males was also lower (27% vs 49 % respectively).

### Cohort characteristics and relationships with sex, age and menopausal status

#### Differences in the association of age with metabolic traits between males and females

**Figure 2:**
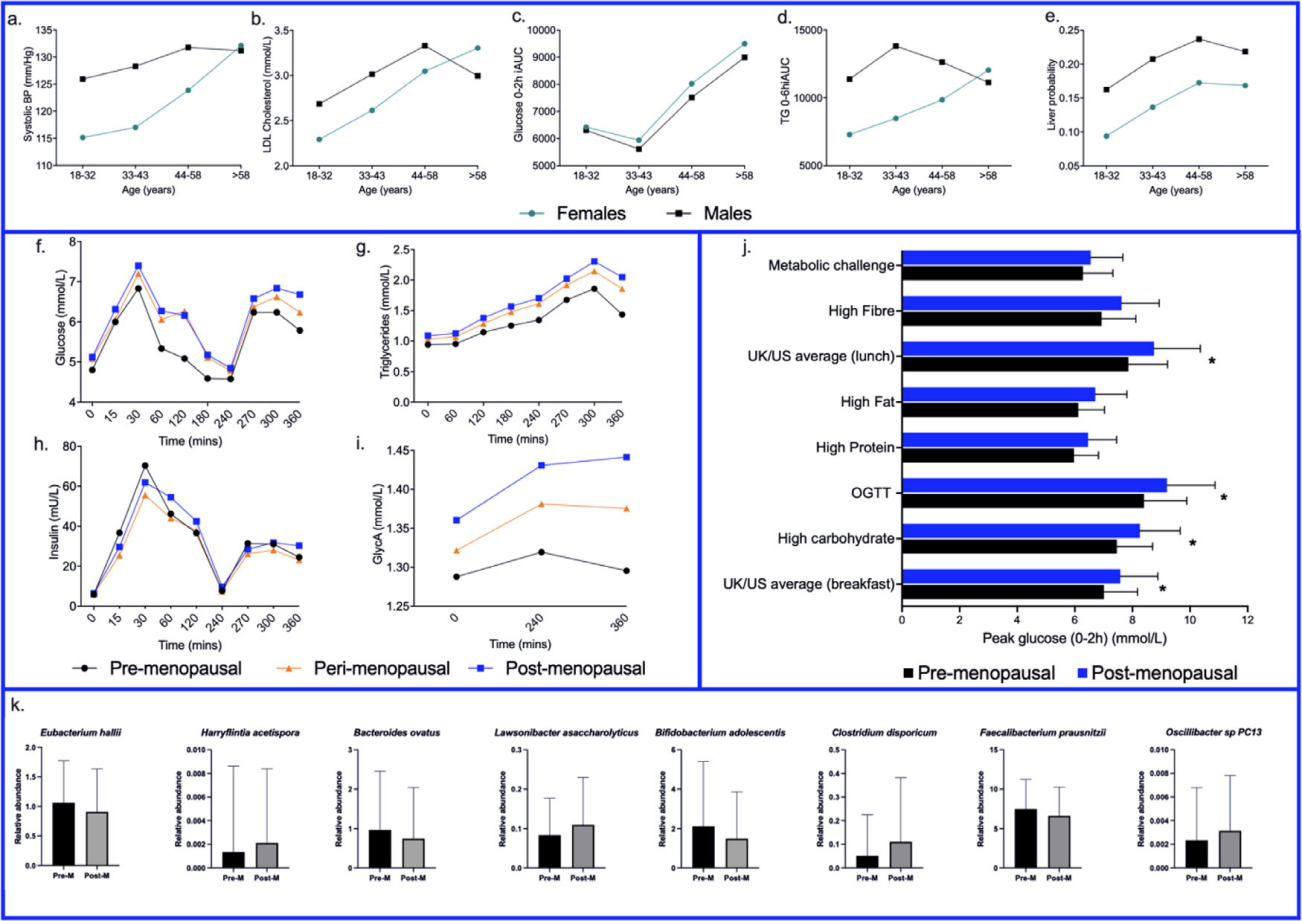
Variation in males and females across age groups. Age groups selected to represent the pre-menopausal period (18–32y and 33–43y, pre-menopausal females only), the menopausal transition period (44–58y, includes pre-, peri- and post-menopausal females) and the post-menopausal period (>58y, post-menopausal females only) **a.** Systolic BP (mm/Hg) **b.** LDL-C (mmol/L), **c.** Glucose_2hiauc_, **d.** Triglyceride_6hiauc__,_**e**. Liver probability score. Black dots and lines represent males (*N*=273; 18–32y, *n*=68; 33–43y, *n*=64; 44-58y, *n*=110; >58y, *n*=32), green dots and lines represent females (*N*=725; 18–32y, *n*=111; 33–43y, *n*=153; 44-58y, *n*=339; >58y, *n*=122). Variation in clinic postprandial metabolic responses for **f**. Glucose, **g**. TG, **h.** Insulin and **i.** GlycA in pre- (n=365), peri- (*n*=55) and post-menopausal (*n*=206) females. Lines represent average values for pre- (black), peri- (orange) and post-menopausal (blue) females. **j.** CGM-derived peak glucose (0–2 h) concentrations following 8 set meals (metabolic challenge, high fibre, high fat, high protein, high carbohydrate, oral glucose tolerance test, US/UK representative (breakfast and lunch) meals) for pre (*n*=365) and post-menopausal (*n*=206) females. k. Gut bacterial species with significantly different relative abundances (n species=8) between pre- *vs.* post-menopause females (*n*=564) in the PREDICT 1 cohort.

We first characterised the role of age and sex in cardiometabolic health, anthropometry, lifestyle and diet measures (Table 1). Males had more unfavourable body composition, fasting and postprandial blood profiles and a lower diet quality than females (*p*<0.05, after age and BMI adjustment (ANCOVA)). When associations with age were examined across all measures for both males and females, several markers were significantly associated with age in females but not in males, including blood pressure (BP), inflammation, fasting LDL-C, TG and insulin and postprandial TG and glucose (systolic BP (SBP); *r*=0.35, GlycA (a measure of inflammation); *r*=0.09, TG; *r*=0.11, insulin; *r*=−0·09, LDL-C; *r*=0.42, glucose_2hiauc_; *r*=0.21 and triglyceride_6hiauc_; *r*=0.22), whereas the opposite was seen for liver probability scores, which estimates the presence of fatty liver (males; *r*=−0.16 *vs.* females; *r*=0.08) (Figure 2 a-e). These relationships persisted in a subcohort of females (apart from TG, insulin and GlycA), matched in sample size (*n*=274, mean of multiple random sub samples reported) to the male cohort.

### Associations with menopausal status

Given the divergent age-sex response, we then characterised the female cohort according to pre-, peri- and post-menopausal status (adjusting for age, BMI, MHT use and smoking status). Following the menopause transition, post-menopausal females were significantly older (mean difference 19.9 years, 95% CI; 18.7–21.0), had higher SBP (12.2 mm/Hg, 95% CI; 9.6–14.8), had unfavourable fasting blood concentrations (higher glucose; 0.30 mmol/L, 95% CI; 0.23–0.39, insulin; 0.44 mmol/L, 95% CI; 0.30–1.19, GlycA; 0.07 mmol/L, 95% CI; 0.05–0.10 and glycosylated haemoglobin (HbA1c); 0.22%, 95% CI; 0.18–0.20), higher insulin insensitivity (HOMA-IR; 0.20, 95% CI; 0.01–0.40) and higher ASCVD 10y risk (0.02, 95% CI; 0.02–0.03) (Table 2). No significant differences were observed in fasting lipoprotein size, concentration or composition from NMR metabolomic analysis after FDR adjustment (Supplementary Table 3). Post-menopausal females also reported greater sleep difficulties (higher Pittsburgh Sleep Quality Index; *p*<0.001) and had higher dietary intakes of total sugar explained by higher intakes of sweets and desserts (*p*<0.05) (ANCOVA) (Supplementary Table 4). Although BMI was greater and physical activity lower in post- *vs.* pre-menopausal females it was not statistically significant. Visceral fat mass, whole body fat percentage and bone mineral density, as measured by DEXA, were also not different between groups. Differences remained in the same direction between pre- and post-menopausal females when stratified according to BMI; Supplementary Table 5.

**Table 2:** Characteristics of the PREDICT 1 cohort across menopausal status.

	Total menopausal cohort+		Pre-menopause		Peri-menopause		Post-menopause		Pre-M vs post-M	Pre-M vs peri-M
	Mean (SD)	n	Mean (SD)	n	Mean (SD)	n	Mean (SD)	n	*p*-value	*p*-value
Age (years)	46.4 (12.0)	(619)	38.6 (9.05)	(359)	52.3 (3.48)	(55)	58.5 (5.05)	(205)	0.00***	0.00***
Systolic BP (mm/Hg)	123 (14.8)	(613)	118 (12.4)	(359)	126 (12.8)	(54)	130 (16.1)	(200)	0.00***	0.14
Diastolic BP (mm/Hg)	75.4 (10.0)	(613)	73.7 (10.24)	(359)	77.3 (9.30)	(54)	78.0 (9.12)	(200)	0.33	0.70
**Body composition**										
Height (cm)	165 (6.44)	(619)	166 (6.38)	(359)	165 (6.34)	(55)	164 (6.35)	(205)	0.11	0.96
Weight (kg)	69.3 (14.1)	(619)	68.1 (13.77)	(359)	72.9 (16.2)	(55)	70.3 (13.7)	(205)	0.09	0.96
BMI (kg/m^2^)	25.4 (5.32)	(619)	24.8 (5.01)	(359)	26.7 (5.87)	(55)	26.3 (5.53)	(205)	0.26	0.96
Visceral fat mass (g)	505 (315)	(569)	414 (257)	(330)	580 (393)	(50)	645 (330)	(189)	0.37	0.70
Waist: Hip ratio	0.82 (0.07)	(619)	0.80 (0.07)	(359)	0.83 (0.07)	(55)	0.84 (0.07)	(205)	0.29	0.70
**Fasting blood markers**										
Glucose (mmol/L)	4.94 (0.53)	(619)	4.81 (0.42)	(359)	5.08 (0.94)	(55)	5.12 (0.48)	(205)	0.00***	0.71
Triglycerides (mmol/L)	1.00 (0.49)	(619)	0.94 (0.47)	(359)	1.04 (0.54)	(55)	1.09 (0.48)	(205)	0.19	0.71
Insulin (mIU/L)	6.08 (4.17)	(619)	5.94 (3.84)	(359)	5.88 (4.49)	(55)	6.38 (4.61)	(205)	0.00***	0.73
GlycA (mmol/L)	1.31 (0.18)	(619)	1.29 (0.17)	(359)	1.32 (0.2)	(55)	1.36 (0.17)	(205)	0.00***	0.70
Cholesterol (mmol/L)	4.94 (0.98)	(619)	4.61 (0.86)	(359)	5.22 (0.83)	(55)	5.43 (0.98)	(205)	0.03*	0.53
LDL (mmol/L)	2.85 (0.78)	(608)	2.60 (0.7)	(356)	3.03 (0.59)	(54)	3.25 (0.8)	(198)	0.19	0.79
Non-HDL (mmol/L)	3.18 (0.99)	(619)	2.94 (0.9)	(359)	3.40 (0.83)	(55)	3.55 (1.05)	(205)	0.85	0.79
HbA1c (%)	5.49 (0.35)	(618)	5.40 (0.26)	(358)	5.54 (0.71)	(55)	5.62 (0.28)	(205)	0.03*	0.70
Quicki score	0.38 (0.06)	(619)	0.39 (0.06)	(359)	0.40 (0.08)	(55)	0.38 (0.05)	(205)	0.03*	0.79
HOMA-IR	1.37 (1.08)	(619)	1.29 (0.9)	(359)	1.41 (1.46)	(55)	1.50 (1.23)	(205)	0.00***	0.66
ASCVD 10y risk	0.01 (0.02)	(251)	0.01 (0.01)	(154)	0.02 (0.01)	(22)	0.03 (0.02)	(75)	0.00***	0.08
Liver Fat Probability score	0.15 (0.13)	(565)	0.13 (0.12)	(331)	0.20 (0.16)	(53)	0.17 (0.14)	(181)	0.53	0.08
**Postprandial markers**										
Glucose_2hiauc_ (mmol/L.s)	7699 (5273)	(551)	6579 (4247)	(318)	8883 (6025)	(50)	9322 (6132)	(183)	0.05*	0.53
Insulin_2hiauc (_mIU/L.s)	277060 (179846)	(494)	277806 (185712)	(317)	229482 (117331)	(51)	288960 (182475)	(184)	0.03*	0.71
Triglyceride_6hiauc_ (mmol/L.s)	9616 (7177)	(552)	8517 (6601)	(286)	10736 (9048)	(41)	11222 (7312)	(167)	0.5	0.57
GlycA_6rise_	-0.02 (0.03)	(589)	-0.02 (0.03)	(345)	-0.02 (0.02)	(51)	-0.02 (0.03)	(193)	0.65	0.70
**Lifestyle & diet**										
Sleep (PSQI, 0-21)	6.90 (2.28)	(575)	6.61 (2.29)	(342)	7.04 (2.05)	-51	7.42 (2.23)	-182	0.00***	0.24
Activity level (0-5)	3.78 (1.61)	(618)	3.85 (1.51)	(359)	4.07 (1.79)	(54)	3.57 (1.71)	(205)	0.35	0.71
Diet (HEI, 0-100)	59.1 (10.2)	(564)	59.8 (10.1)	(335)	58.8 (11.5)	(49)	57.8 (9.77)	(180)	0.16	0.92
Energy (kcal)	1623 (468)	(564)	1580 (466)	(335)	1678 (511)	(49)	1688 (452)	(180)	0.19	0.70
Carbohydrate (% energy)	43.9 (8.11)	(563)	44.4 (8.22)	(335)	43.4 (8.4)	(49)	43.0 (7.78)	(180)	0.07	0.53
Protein (% energy)	18.1 (3.40)	(563)	17.9 (3.46)	(335)	18.9 (3.38)	(49)	18.3 (3.27)	(180)	0.37	0.96
Fat (% energy)	37.2 (6.30)	(563)	37.3 (6.29)	(335)	37.2 (6.59)	(49)	37.2 (6.28)	(180)	0.19	0.53
Sugars (g)	83.8 (33.4)	(564)	80.0 (32.9)	(335)	89.6 (35.3)	(49)	89.4 (33.0)	(180)	0.00***	0.08
**Mood**										
Less accomplished (Yes%)	19.3	(613)	23.3	(356)	18.2	(55)	14.4	(202)	0.92	0.70
Less concentration (Yes%)	11.3	(609)	13.6	(354)	1.82	(55)	9.00	(200)	0.37	0.53
Contentedness*	3.01 (1.09)	(614)	3.17 (1.09)	(357)	2.78 (1.03)	(55)	2.80 (1.08)	(202)	0.85	0.53
Energetic*	3.11 (1.18)	(614)	3.25 (1.19)	(357)	2.93 (1.17)	(55)	2.92 (1.15)	(202)	0.19	0.32
Sadness*	4.87 (1.00)	(614)	4.75 (1.02)	(358)	5.07 (0.86)	(55)	5.03 (0.98)	(201)	0.03*	0.66
Lower social activity*	4.56 (0.74)	(616)	4.45 (0.80)	(358)	4.71 (0.60)	(55)	4.72 (0.62)	(203)	0.49	0.57

*Scoring ranges from lowest “all of the time” to highest “none of the time”. ANCOVA (continuous) and logistic regression (categorical) adjusted for age and BMI. Anthropometric traits were adjusted for age only. Differences between menopausal groups also adjusted for MHT use and smoking status. All p-values adjusted for multiple testing (FDR<0.05); * *p*<0.05, ** *p*<0.01, ***p<0.001.+ PREDICT 1 females who self-reported menopausal status.

### Menopausal status associations with postprandial responses and glycaemic variability

Postprandial elevations in circulating lipids and glucose are associated with increased risk of cardiometabolic disease, type-2 diabetes and obesity, independent from fasting measures.^9^ Therefore, as humans spend the majority of their day in the postprandial metabolic state, we next examined differences in postprandial responses between pre- and post-menopausal females (adjusting for age, BMI, MHT use and smoking status). Clinic postprandial metabolic responses differed between groups (Figure 2 f-i), with significantly higher glucose_2hiauc_ and insulin_2hiauc_ (*p*<0.05) (ANCOVA) in post- *vs.* pre-menopausal females.

Given the differences in postprandial glycaemia measured in the tightly controlled clinic setting, we then explored glycaemia in the remote phase of the study using CGM data. We examined different features of glycaemic responses, including glycaemic variability (measured by coefficient of variation), time spent in range, mean day long glucose concentration, as well as glucose_2hiauc_ and peak_0-2h_ following meals of varying macronutrient composition. Mean day-long glucose concentrations and glycaemic variability (examined using 2-4 free-living days of the PREDICT 1 remote phase) were higher in post-menopausal females (5.1±0.53 mmol/L and 17.6±4.3 %) compared to pre-menopausal females (4.9±0.54 mmol/L and 15.6±4.00 %), *p*<0.002 (ANCOVA) (Supplementary Table 6). Pre-menopausal females also elicited a more favourable TIR (3.9–5.6 mmol/L) (70.8%±16.9) compared with post-menopausal females (68.8%±15.6), *p*<0.05 (ANCOVA).

Glucose_2hiauc_ and glucose peak_0-2h_ were next examined across seven isocaloric meals differing in protein, fat, carbohydrate, and fibre content, as well as an oral glucose tolerance test (OGTT) following an overnight fast (nutritional composition of meals available in Supplementary Table 1). For all meals, postprandial glucose concentrations (glucose_2hiauc_ and peak_0-2h_) were higher in post- *vs*. pre-menopausal females (Supplementary Table 7), with the greatest differences observed following a nutrient composition reflective of a typical UK/US average diet (composition 40% fat and 57% carbohydrate; mean difference in glucose_2hiauc_ 3524.3 and peak_0-2h_ 0.89 mmol/L, *p*<0.05) followed by the OGTT (glucose_2hiauc_ 2598.2 and peak_0-2h_ 0.80 mmol/L) and high carbohydrate meal (glucose_2hiauc_ 2291.5 and peak_0-2h_ 0.79 mmol/L). Glycaemic responses (glucose_2hiauc_) remained significantly higher in post-menopausal females for the typical UK/US average meal and (peak_0-2h_) for high carbohydrate meal, typical UK/US average meals and OGTT, after correcting for covariates compared with pre-menopausal females (*p*<0.05) (Figure 2j and Supplementary Table 7). Postprandial lipoprotein profiles (NMR analysis) were not significantly different (FDR adjusted).

Menopausal status also corresponded to a state of greater inter-individual variability (coefficient of variation) in post- *vs*. pre-menopausal females for postprandial insulin (30 min, pre-menopause 89% *vs.* post-menopause 200%) and HOMA-IR (pre-menopause 69.4% *vs.* post-menopause 82.6%) (Supplementary Figure 1 and Supplementary Table 8).

### Menopausal status associated with microbiome composition

We have previously reported a strong association between gut microbiome composition and metabolic health (both fasting and postprandial measures).^7^ Therefore, we examined gut microbiome composition, within-sample richness (number of species) and within-sample diversity (Shannon) in pre- and post-menopausal females (*n*=564). The relative abundances of microbiome species differed with menopausal status, with eight species significantly differentially abundant after correction for multiple testing (*p*<0.2) (Mann-Whitney U test) (Supplementary Table 9 and Figure 2k). Of these, four species had significantly higher abundances in pre- *vs.* post-menopausal females, whereas four species had higher abundances post-menopause (*p*<0.2). *Bacteroides ovatus* has been associated with younger age in a large meta-analysis,^12^ but none of the other species were associated with age or BMI. Microbiome richness and diversity were not significantly different (Supplementary Table 9).

### Is the association of menopause with metabolic traits independent of age?

**Figure 3:**
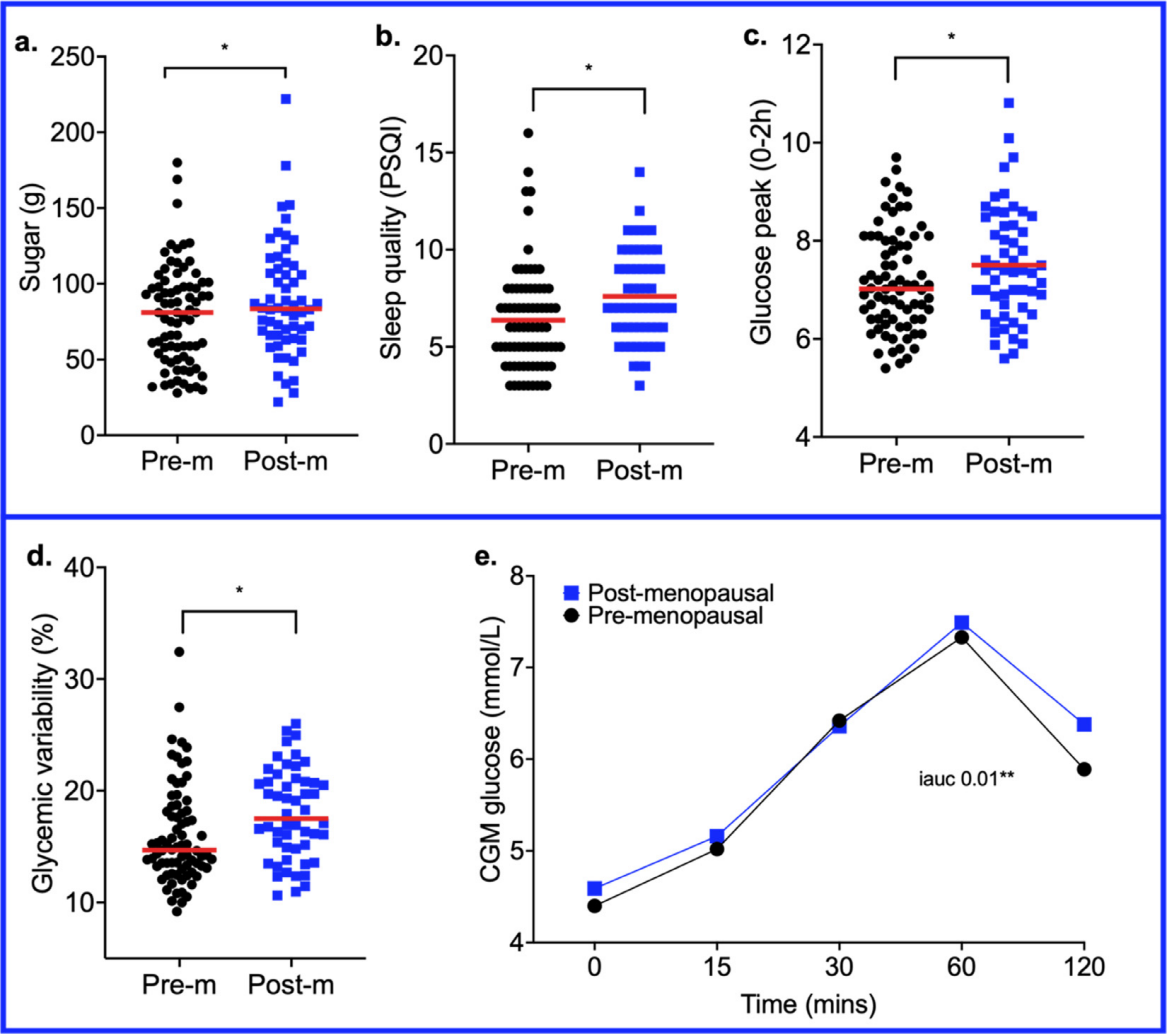
Distribution of a. sugar intake, b. sleep quality, c. postprandial glucose peak_0-2h_ and d. glycaemic variability between age-matched pre- (*n*=86) and post-menopausal (*n*=64) females. The red line represents the group median. **e.** Variation in postprandial glucose responses (0–2 h) to a typical UK/US average lunch, measured by CGM. Pre-m; pre-menopausal, Post-m; post-menopausal.

Menopausal status is an age-related biological event; thus to untangle some of the effects of age from menopausal status we examined cardiometabolic health, anthropometry, lifestyle and diet measures in an age-matched subgroup of pre- and post-menopausal females. Measures that were significantly different with menopausal status in the total cohort were examined (adjusted for age, BMI, MHT use and smoking status). Sleep and intakes of sugar remained significantly different in the age-matched subgroup (*n*=150, age range 47–56y) (Figure 3a-b) (Supplementary Table 10). Postprandial glycaemic measures also remained significantly higher in post- *vs.* pre-menopausal females (*p*<0.05), (Supplementary Table 11) for clinic postprandial glucose peak_0-2h_ (7.62±1.15 mmol/L *vs.* 7.19±1.03 mmol/L (Figure 3c)); CGM glycaemic variability (17.9±4.1 % *vs.* 15.9±4.4 %) (Figure 3d) and glucose_2hiauc_ (150558±5384 *vs.* 13258±5657; following meal reflective of a typical UK/US average diet) (Figure 3e).

When investigating differences in microbiome composition, in the age matched cohort, the abundances of six species were significantly different between pre- and post-menopausal females but were not significant after FDR correction (Supplementary Table 9). From the eight species previously identified in the total cohort, four species showed the same directional trend but were not significant after FDR correction (*Eubacterium hallii, Bifidobacterium adolescentis, Faecalibacterium prausnitzii, Oscillibacter sp PC13*). The remaining four species, (*Harryflintia acetispora, Bacteroides ovatus, Lawsonibacter asaccharolyticus* and *Clostridium disporicum)* were not abundant in the same directional trend in the age-matched subgroup.

Due to the deficiency in female sexual hormones observed post-menopause we also examined measures in age-matched 1) males, 2) pre-menopausal females and 3) post-menopausal females (age range 47–56y, Supplementary Table 10). Females, both pre- and post-menopausal, had significantly lower SBP and ASCVD 10y risk compared to males. However, pre-menopause was associated with more favourable fasting blood glucose and GlycA concentrations, and mean day long glucose concentrations (CGM) compared to males (*p*<0.05) (ANCOVA), while male levels were more similar to post-menopausal females. Interestingly, glycaemic variability, which worsened post-menopause (compared with age-matched pre-menopausal females), was also significantly higher than males (*p*=0.007) (ANCOVA), suggesting unfavourable blood glucose variability independent of age and gender.

### The association between post-menopausal hormone therapy use and metabolic health

MHT is commonly used among females undergoing the menopausal transition. Given the replacement of vital female sexual hormones, most notably oestrogen, by MHT and the involvement of these in gut-metabolic interactions,^13^ we examined the link between MHT use and metabolic health in post-menopausal females (Table 3; adjusted for age, BMI and smoking status). Measures that were significantly different with menopausal status in the total cohort were examined along with a selection of measures with previously known associations with MHT (visceral fat and lipids (LDL-C and triglyceride_6hiauc_)).^14^ Post-menopausal females using MHT (*n*=35) had lower visceral fat mass, favourable fasting blood concentrations (for glucose, insulin, cholesterol (total and LDL)), and lower postprandial lipaemia (triglyceride_6hiauc_) compared to non-MHT users (*n*=172), *p*<0.05 for all (Table 3). Furthermore, to disentangle the effect of genetics, we examined our predominantly twin cohort and identified six post-menopausal MZ twin pairs discordant for MHT use. HbA1c showed significant differences between discordant twin pairs (*p*=0.004) (Paired t-test) (Supplementary Table 12). We observed a more favourable body composition (lower BMI, weight, visceral fat) and blood biomarker concentrations (lower glucose, insulin, TG and GlycA) in twins using MHT *vs.* those non-MHT users (although not significant).

**Table 3:** Menopausal hormone therapy use in post-menopausal females.

	Post-menopausal taking MHT			Post-menopausal not taking MHT			MHT vs no MHT
	n	mean	SD	n	mean	SD	*p*-value
Age (years)	35	57.9	5.35	172	58.7	4.97	0.35
Systolic BP (mm/Hg)	35	130	13.0	165	131	16.7	0.69
*Body composition*							
Visceral fat mass (g)	33	537	292	157	665	335	0.03*
*Fasting blood biomarkers*							
Glucose (mmol/L)	35	4.90	0.46	172	5.17	0.47	0.01**
Triglycerides (mmol/L)	35	1.10	0.48	172	1.09	0.48	0.39
Insulin (mIU/L)	35	4.76	2.84	172	6.71	4.82	0.04*
GlycA (mmol/L)	35	1.33	0.17	172	1.37	0.16	0.38
Cholesterol (mmol/L)	35	5.10	0.74	170	5.49	1.01	0.03*
LDL (mmol/L)	35	2.95	0.65	163	3.31	0.81	0.03*
HbA1c (%)	35	5.54	0.27	170	5.64	0.28	0.15
Quicki score	35	0.40	0.04	170	0.38	0.05	0.04*
HOMA IR	35	1.05	0.67	170	1.59	1.30	0.03*
ASCVD 10y risk	14	0.03	0.02	61	0.03	0.02	0.99
**Postprandial markers**							
Glucose_2hiauc_ (mmol/L.s)	33	9726	5120	149	9241	6366	0.42
Insulin_2hiauc_ (mIU/L.s)	33	296783	185927	150	287587	182859	0.43
Triglyceride_6hiauc_ (mmol/L.s)	30	8880	6382	136	11777	7434	0.04*
**Lifestyle**							
Sleep (PSQI, 0-21)	31	8.06	2.49	153	7.31	2.15	0.05

*Post-menopausal females who self-reported currently taking MHT.

### Mediating effects of sleep, physical activity, diet and microbiome on the link between menopausal status and key metabolic health indicators

**Figure 4:**
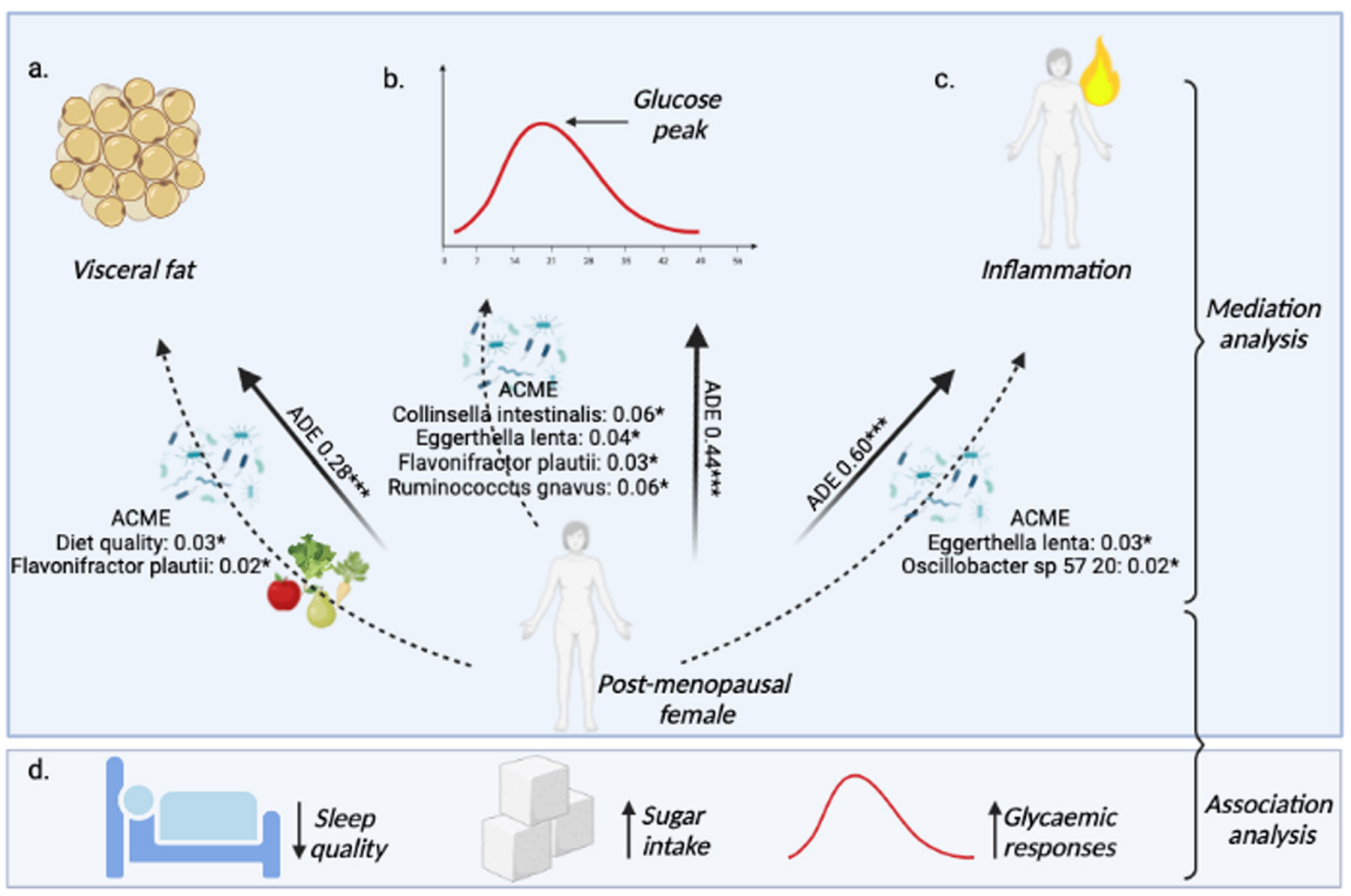
Mediation analysis of the association between menopausal status and **a.** visceral fat (g) **b.** glucose (peak_0-2h_) **c.** GlycA (mmol/L). Average direct effect (ADE) and average causal mediation effects (ACME) are reported (*P<0.05, **P < 0.01, ***P < 0.001). d. Health and diet measures associated with post-menopausal status.

Changes to lifestyle, diet, mood, anthropometry, gut and cardiometabolic health following the menopause transition are intricately linked. We hypothesised that key pillars of health, including sleep, physical activity, diet and gut health, may mediate the adverse metabolic effects observed post-menopause for several health outcomes. Thus we conducted a formal mediation analysis to determine the mediating effect of sleep, physical activity, diet quality (Healthy Eating Index (HEI)) and microbiome species to assess the link between menopausal status and key metabolic health indicators (visceral fat, inflammation (GlycA_360mins_) and glycaemia (peak_0-2h_)). Diet quality, in part, mediated the association between menopause status and visceral fat (proportion mediated; 9%, *p*<0.05) (Figure 4a and Supplementary Table 13). For the microbiome species, we examined the eight species that were different between pre- and post-menopausal females in the total cohort (Figure 2k) and the 30 species previously associated with cardiometabolic health and diet in this cohort.^7^ Four microbiome species (*Collinsella intestinalis, Eggerthella lenta, Flavonifractor plautii* and *Ruminococcus gnavus)* acted as partial mediators in the association between menopause status and GlycA (proportion mediated; 5–10%) (Figure 4b and Supplementary Table 13). The species *Flavonifractor plautii and Eggerthella lenta* acted as a partial mediators in the association between menopause status and visceral fat (proportion mediated; 5%) and glucose (peak_0-2h_) (proportion mediated; 8%, *p*<0.05) respectively. Conversely, *Oscillibacter sp 57 20* acted as partial mediators in the association between menopause status and glucose (peak_0-2h_) (proportion mediated; −6%) (Figure 4c) (*p*<0.05 for all).

## Discussion

With our ageing population, it is estimated that worldwide 1.2 billion women will be in the menopausal transition or post-menopause by the year 2030.^15^ In the current study, we demonstrate that post-menopause status is associated with unfavourable changes in body composition, fasting and postprandial blood profiles (including inflammation and postprandial glycaemia), diet, sleep and gut microbiome. We further explored the independent role of menopause from age and observed poorer sleep and diet, as well as higher postprandial glycaemic measures post-menopause, alongside a protective association of MHT use with visceral fat, fasting (glucose and insulin) and postprandial (triglyceride_6hiauc_) blood measures. Finally, we investigated the association between modifiable risk factors on metabolic changes in menopause, finding a mediating effect of diet and a gut bacterial species and visceral fat, glycaemia and inflammation, by menopause status. Changes in key metabolic health indicators observed in menopause may therefore be attenuated by targeting the gut microbiome and diet.

Differences in typically measured features of the postprandial responses for glucose and TG have previously been reported in pre- versus post-menopausal females in small studies looking at single postprandial measures.^6^^,^^16^^,^^17^ Here, we show that in addition to 2h iAUC for glucose and insulin, post-menopausal females had a more unfavourable glycaemic variability, TIR and day-long glucose, measured by CGM in free-living days. Continuous glycaemic features capture day-to-day glycaemic excursions, including peak concentration, nadirs ‘below baseline’, glycaemic variability and TIR, each associated with downstream metabolic effects, including oxidative stress, inflammation, and increased cardiovascular and diabetes disease risk.^18^ In this cohort, we did not see differences in postprandial TG independent of age. To the best of our knowledge, one previous study has compared postprandial TG response between pre- and post-menopausal females using a sequential meal postprandial investigation.^17^ Females were subdivided into both younger and older pre- and post-menopause subgroups but no differences in postprandial TG were observed due to menopausal status.^17^ Differences were observed between young and old pre-menopausal groups, suggestive that major increases observed in postprandial TG may occur during the later pre-menopause years.^17^ This study highlights that inherent biological differences exist between males and females while also demonstrating the protective effects of oestrogen through the controlled comparison of age-matched males and pre- and post-menopausal females. Multiple aspects of health and glucose homeostasis are regulated differently between sexes, and our findings highlight the added complexity introduced by the female menopausal transition.

The protective association of MHT use post-menopause with metabolic measures, further supports an independent effect of oestrogen observed in existing research. Favourable effects on visceral fat mass and fasting blood concentrations and lower postprandial lipaemia post-menopause are in accordance with evidence that MHT can provide effective relief in low-risk females for a wide range of adverse health outcomes and symptoms associated with menopause.^14^^,^^19^ However, MHT is available in multiple forms and doses, and the impact of different therapies is beyond the scope of this research. For example, transdermal oestradiol and micronised progesterone are not associated with a risk of venous thromboembolism compared to oral oestrogen with a synthetic progestogen.^20^ Given the appropriate dose and type, MHT can reduce biological ageing and provide effective treatment to alleviate menopausal symptoms and confer protective cardiometabolic effects in appropriate candidates.^20^

Female sex hormones influence the microbiota at multiple body sites, including the gut,^13^ which has been associated with multiple diseases outside of this organ. The gut microbiota metabolizes oestrogen-like compounds consumed in plant foods (phytoestrogens), including lignans (derived from a variety of plant foods) and isoflavonoids (found in soy foods).^21^^,^^22^ Administration of isoflavone-rich soy foods to post-menopausal females can lead to elevated levels of gut microbial derived oestrogen-like metabolites^21^ and changes in some bacteria including increases in *Bifidobacterium* and decreases of *Clostridiaceae*, which play roles in inflammatory diseases.^22^ Further, changes in oestrogen receptor (ER-β) expression have been shown to affect microbiota composition^23^ and the gut microbiomes of pregnant females were profoundly altered during the third trimester, when oestrogen is at its peak.^24^ Thus a reciprocal relationship may exist between oestrogen and the microbiome which may modulate the health of menopausal females. Our findings show differences in abundances of species post-menopause, including pro-inflammatory and obesogenic bacteria. Of interest, four species in part modulated the relationship between menopause and GlycA, a marker of inflammation. Our previous research associated these species with unfavourable cardiometabolic health, diet and inflammatory outcomes,^7^ in line with previous research.^25^, ^26^, ^27^ For example, *Ruminococcus gnavus*, a prevalent gut microbe, produces a potent, inflammatory polysaccharide recognized by innate immune cells through the toll-like receptor 4 (TLR4) and is associated with multiple inflammatory diseases.^25^ GlycA is strongly associated with cardiovascular and diabetes risk^28^; thus, our data suggests that inflammation may be reduced through intervention aimed at improving the gut microbiome post-menopause.

The mediating effects of diet and microbiome on visceral fat, glyceamia and inflammation respectively, suggest that modifiable factors may play a role in the unfavourable changes observed post-menopause. Given that many diet and lifestyle changes also occur during the menopausal transition,^29^ which are known to impact metabolic health outcomes, these are potential targets to alleviate some of the downstream unfavourable health effects associated with menopause. For example, in our cohort, post-menopausal females consumed higher intakes of dietary sugars and reported poorer sleep. These are both associated with more pronounced postprandial glycaemia^30^ and increased risk of type-2 diabetes and cardiovascular disease.^31^ Further, a decrease in physical activity energy expenditure and a shift to a more sedentary lifestyle associated with menopause^32^ was recently demonstrated to be a direct effect of declining oestrogen^33^ which may have been captured with more quantitative measures of physical activity in this study. These observed changes in diet and physical activity may increase the risk for positive energy balance and weight gain over time. Healthy dietary patterns such as the Mediterranean diet have been associated with improved weight and vasomotor symptoms in post-menopausal females^34^ and certain foods have also been linked to later onset of menopause i.e. intakes of green and yellow vegetables as well as fresh legumes.^35^ Positive health effects associated with diet quality, may be due to higher contents of dietary fiber, complex carbohydrates, vitamins, minerals, polyunsaturated fatty acids, and phytochemicals. Diets high in plant-based foods may be naturally rich in isoflavones which may play a role in the protective effects some diet patterns such as Mediterranean diet have on menopause. Research shows that 60% of women consulted healthcare providers during their menopausal transition seeking support for menopausal symptoms and treatment,^36^ highlighting an opportune window to target modifiable factors including diet and lifestyle as well as considering MHT.

Limitations of this cross-sectional analysis include; 1) potential inaccuracy in the self-reported time since menopause and self-identification of menopausal status due to ambiguity in determining amenorrhea; 2) cross-sectional data preventing identification of causal relationships; 3) inability to fully account for age-related effects despite the creation of an age-matched subgroup and assessment of MHT discordance; 4) inability to determine menstrual cycle regularity, current contraception use, MHT type (transdermal *vs*. oral) and other current medication use. However, the data presented links changes in postprandial metabolism, metabolic syndrome factors, mood, sleep, diet and the gut microbiome in a single deeply phenotyped cohort. This may help inform specific hypotheses to design dietary intervention studies examining the impact of menopause status on postprandial metabolism and microbiome composition.

In summary, the physiological effects of menopause are numerous and the menopause transition is a time of great change and unfavourable metabolic effects. Whilst this transition is inevitable, this analysis demonstrates that approaches can be taken to attenuate the adverse cardiometabolic sequelae, including a focus on modifiable factors, such as diet, microbiome and use of MHT in appropriate candidates.

## Contributors

Study design and developed concept: S.E.B, A.M.V, J.W, G.H, R.D, N.S, P.W.F, T.D.S. Data collection: S.E.B, I.L, J.W, G.H, T.D.S. Data analysis: K.M.B, F.A. Study coordination: S.E.B, J.W, I.L, G.H, T.D.S. Writing the manuscript: K.M.B, S.E.B, A.M.V, W.L.H, I.L, K.K, J.W, J.E.M, L.R.N, L.D, J.M.O, A.T.C, T.D.S. Obtained funding: J.W, G.H, T.D.S. Accessed and verified the data: K.M.B, F.A, S.E.B, A.M.V. Decided to submit the manuscript: S.E.B and K.M.B. All authors read and approved the final manuscript.

## Data sharing statement

The data used for analysis in this study are held by the Department of Twin Research at King's College London and access can be requested from https://twinsuk.ac.uk/resources-for-researchers/access-our-data/ to allow for anonymisation and compliance with GDPR standards.

## Declaration of interests

TDS, JW and GH are co-founders of ZOE Ltd (ZOE). AMV, FA, LMD, NS, PWF, SEB, TDS receive payments as consultants to ZOE. GH, IL, JW, KK, RD are employed by ZOE. AMV, GH, IL, JW, KK, LMD, NS, PWF, RD, SEB, TDS also receive options in ZOE. Other authors have no conflict of interest to declare.

## Appendix Supplementary materials

Image, application 1

Image, application 2

Image, application 3
