# Supplementary material for: Menopause is associated with postprandial metabolism, metabolic health and lifestyle: The ZOE PREDICT study
Source: eBioMedicine. 2022 Oct 18;85:104303. doi: 10.1016/j.ebiom.2022.104303 (PMC9669773; doi:10.1016/j.ebiom.2022.104303)
Supplement: Supplementary file 1 [file mmc1.docx]

Supplementary Materials

Supplementary Tables

| List of supplementary tables |  |
| --- | --- |
| Supplementary Table 1 | Test meal nutritional composition |
| Supplementary Table 2 | Characteristics related to menopausal status |
| Supplementary Table 3 | Descriptives of NMR metabolites at fasting and postprandially (360 mins) in the total cohort and age-matched subgroup |
| Supplementary Table 4 | Dietary intakes for the pre-, peri- and post-menopausal females in the PREDICT 1 cohort |
| Supplementary Table 5 | Characteristics of pre, peri and post-menopausal females in PREDICT 1 cohort stratified according to BMI groups |
| Supplementary Table 6 | Glycaemic variability and time in range in the PREDICT 1 cohort |
| Supplementary Table 7 | Glycaemic responses to test meals |
| Supplementary Table 8 | Inter-individual variability in body composition, fasting blood biomarkers, lifestyle factors, diet and postprandial measures |
| Supplementary Table 9 | Differences in the relative abundances of gut microbiome species due to menopause status in total cohort and age-matched subgroup |
| Supplementary Table 10 | Characteristics of pre and post-menopausal females and males in the age-matched subgroup |
| Supplementary Table 11 | Association between menopausal status and postprandial metabolic responses in age matched pre- and post-menopausal females |
| Supplementary Table 12 | Post-menopausal MZ twins discordant for MHT use |
| Supplementary Table 13 | Mediation analysis of the association with sleep, physical activity, diet and microbiome species between menopausal status and key metabolic health indicators |
