# Supplementary material for: Menopause is associated with postprandial metabolism, metabolic health and lifestyle: The ZOE PREDICT study
Source: eBioMedicine. 2022 Oct 18;85:104303. doi: 10.1016/j.ebiom.2022.104303 (PMC9669773; doi:10.1016/j.ebiom.2022.104303)
Supplement: Supplementary file 2 [file mmc2.docx]

**Supplementary Materials**

**Menopause is associated with postprandial metabolism, metabolic health and lifestyle: The ZOE PREDICT study**

Authors: Kate M. Bermingham, Inbar Linenberg, Wendy L. Hall, Kirstin Kadé, Paul W. Franks, Richard Davies, Jonathan Wolf, George Hadjigeorgiou, Francesco Asnicar, Nicola Segata, JoAnn E. Manson, Louise R. Newson, Linda M. Delahanty, Jose M. Ordovas, Andrew T. Chan, Tim D. Spector, Ana M. Valdes, Sarah E. Berry

**Methods**

*Outcome measurements.*

A detailed description of the methods for biochemical and physiological analysis is available elsewhere^3^. Participants arrived at the clinic following a 12h fast and were cannulated for venous blood collection throughout the day, at fasting and 9 postprandial time points (15, 30, 60, 120, 180, 240, 270, 300, and 360 min). Plasma glucose and insulin were measured at all time points, serum TG were measured at hourly intervals (assays were performed by Affinity Biomarkers Labs, UK) and metabolomics (nuclear magnetic resonance by Nightingale Health using the 2020 platform) was measured at 0, 4 and 6 hours. Dual-energy X-ray absorptiometry (DEXA) scans were used to measure body composition including fat, muscle and bone following standard manufacturer’s recommendations (DXA; Hologic QDR 4500 plus), from which visceral fat mass, total body fat (%) and bone mineral density were selected.

*Standardized test meal challenges.*

Serum glucose, TG, insulin and metabolomics were measured fasting and postprandially following standardized test meals. The nutritional characteristics of these meals, and their consumption protocol, are detailed in **Supplementary Table 1**). In brief, the first set of standardized test meals was consumed by participants at the clinic visit under test conditions and consisted of a high fat-high carbohydrate metabolic challenge breakfast and lunch meal containing 1390kcal combined. During the at-home intervention participants were provided with a further 8 test meals delivered as a 500kcal set of muffins, milkshakes and energy bars and a 300kcal OGTT, which varied in their nutritional composition (ranging from 28-95g carbohydrate, 0-53g fat, and 0-41g protein). Participants were trained during their clinic visit in the meal consumption protocol and consumed their at-home intervention meals at breakfast following overnight fasts and at lunch following a further fasting period from breakfast. Meal order was block randomized and meals were consumed in duplicate or singularly, depending on the meal. Consumed standardized set meals were logged by participants in the specialised study app, and were reviewed by study staff in real-time to assess protocol compliance. Criteria used to assess logged meal accuracy were previously described in Berry et al^9^.

*Food and mood data.*

Self-reported food frequency questionnaires were used to measure habitual diet. UK nutrient intakes were determined using FETA software to calculate macro- and micronutrient data. Submitted FFQs were excluded if more than 10 food items were left unanswered, or if the total energy intake estimate derived from FFQ as a ratio of the subject’s estimated basal metabolic rate (determined by the Harris–Benedict equation) was more than 2 s.d. outside the mean of this ratio (<0·52 or >2·58). Self-reported questionnaires on mood were completed by participants, including the following questions, 1) “During the past 4 weeks, have you accomplished less than you would like at your work or other regular daily activities as a result of any emotional problems (such as feeling depressed or anxious)?”, 2) “During the past 4 weeks, have you not done work or other activities as carefully as usual as a result of any emotional problems (such as feeling depressed or anxious)?”, 3) “How much of the time during the past 4 weeks have you felt calm and peaceful?”, 4) “How much of the time during the past 4 weeks did you have a lot of energy?”, 5) “How much of the time during the past 4 weeks have you felt downhearted and blue?” and 6) “How much of the time during the past 4 weeks has you physical or emotional problems interfered with your social activities?”.

*Physical activity.*

Self-reported questionnaires were used to measure physical activity. Energy expenditure was also measured using a triaxial accelerometer (AX3, activity) fitted by nurses at the baseline visit on the non-dominant hand. Accelerometers were programmed to measure acceleration at 50 Hz with a dynamic range of ±8 g (where g refers to local gravitational force equal to 9·8 ms2). Non-wear periods were defined as windows of at least 1 hour with less than 13 mg for at least 2 out of 3 axes, or where 2 out of 3 axes measured less than 50 mg.

*Microbiome samples.*

DNA was isolated by QIAGEN Genomic Services using DNeasy 96 PowerSoil Pro from all day 0 (baseline) DNA/RNA Shield-fixed microbiome samples as previously described by Asnicar et al^7^. Optical density measurement was done using spectrophotometer quantification (Tecan Infinite 200). Before library preparation and sequencing, the quality and quantity of the samples were assessed using the Fragment Analyzer system (Agilent Technologies) according to manufacturer’s guidelines. Samples with a high-quality DNA profile were further processed. The NEBNext Ultra II FS DNA Module (catalog no. E7810S/L; New England Biolabs) was used for DNA fragmentation, end-repair and A-tailing. For adapter ligation, the NEBNext Ultra II Ligation Module (catalog no. E7595S/L; New England Biolabs) was used. The quality and yield after sample preparation were measured with the Fragment Analyzer system. The size of the resulting product was consistent with the expected size of approximately 500–700 bp. Libraries were sequenced for 300-bp paired-end reads using the Illumina NovaSeq 6000 platform according to the manufacturer’s protocols. The 1.1-nM library was used for flow cell loading. The NovaSeq control software NCS v.1.5 was used. Image analysis, base calling and quality checking were performed with the Illumina data analysis pipeline RTA3.3.5 and bcl2fastq v.2.20. All sequenced metagenomes were quality control edited using the pre-processing pipeline as implemented in <https://github.com/SegataLab/preprocessing>. The metagenomic analysis and taxonomic profiling and quantification of organisms' relative abundances of all metagenomic samples were previously described in Asnicar et al^7^. The microbiome species richness of an individual was estimated from the taxonomic profiles of PREDICT 1 participants. Two alpha diversity measures were computed: the number of species found in the microbiome (‘observed richness’); and the Shannon entropy estimation.

*CGM devices.*

Participants wore the Abbott Freestyle Libre Pro (FSL; Abbott, Abbott Park, IL, US). Monitors were worn on the upper arms and covered with Opsite Flexifix adhesive film (Smith and Nephew Medical Ltd, Hull, England) for improved durability. Subcutaneous tissue interstitial glucose levels were recorded every 15 min, within a range of 2·2-22·2 mmol/L. CGMs were worn by participants for 10-14 days. Participants had 2-4 free-living days during the study period, which varied across cohorts. CGM data for free-living days only were selected, meaning no set meals were consumed on these days. CGM data was collected in coordinated universal time (UTC) and free-living days were adjusted to participant specific time zones. Exclusion criteria for analysis included participants with <2 free-living days, >1 time zone during the free-living period, CGM malfunction as classified by >25 readings at monitor baseline per day or >10% missing reads per day. All glucose outcomes are reported as means with s.d. Glucose variability was measured by the coefficient of variation CV (%), calculated as s.d./mean. TIR was calculated based on an optimised TIR cut-off (TIR_optimised_; 3·9-5·6 mmol/L) created for non-diabetic participants.
